# Impact of Geographical Origin on the Contents of Inorganic Elements and Bioactive Compounds in *Polygonum perfoliatum* L.

**DOI:** 10.3390/molecules30102231

**Published:** 2025-05-21

**Authors:** Yanping Zhang, Liyuan Zhao, Xinsheng Wang, Chenxi Zhang, Haichao Zuo, Di Gao

**Affiliations:** Chemistry and Chemical Engineering School, Henan University of Science and Technology, Luoyang 471023, China

**Keywords:** *Polygonum perfoliatum* L., inorganic elements, bioactive compounds, correlations, geographical origins

## Abstract

This study investigated the correlation between thirteen inorganic elements, five key bioactive compounds, and environmental factors in *Polygonum perfoliatum* L. from fifteen different origins. Analyses were conducted using techniques such as ultrasound-assisted extraction, HPLC, ICP-AES, PCA, and HCA. The results indicate that the geographical origin significantly influences the contents of inorganic elements and bioactive compounds in *Polygonum perfoliatum* L., and a certain correlation exists among elements, compounds, and environmental factors. This research provides a theoretical foundation for the development and utilization of *Polygonum perfoliatum* L.

## 1. Introduction

*Polygonum perfoliatum* L. (*P. perfoliatum*), which belongs to the Polygonaceae family, is an annual herb widely distributed in China, with a concentration in southern regions [1]. Its aerial parts are referred to in the Chinese Pharmacopeia as ‘Gangbangui’, which has been traditionally used for the purposes of clearing heat and detoxifying, promoting diuresis and reducing edema, relieving cough and eliminating phlegm, and treating snake and insect bites and furuncles [2]. *P. perfoliatum* has expectorant, anticancer, and antitussive properties, and its phytochemical profile is complex, consisting of various compounds such as flavonoids, alkaloids, and tannins [3,4,5]. Studies have shown that flavonoids and water-soluble phenolic acids are significant contributors to the medicinal properties of the herb. Caffeic acid and gallic acid, which are the main water-soluble phenolic acids in the herb, possess antiviral, anti-inflammatory, and antioxidant properties [6]. On the other hand, isorhamnetin, hyperoside, and quercetin, which are the main flavonoids in the herb, exhibit anti-inflammatory and antitumor properties [2]. As per the Chinese Pharmacopeia guidelines, the level of quercetin is used as an evaluation parameter for *P. perfoliatum* quality [1]. *P. perfoliatum* contains not only bioactive compounds but also inorganic elements. These elements play crucial roles in plants [7]. Developing an understanding of the correlation between the bioactive compounds and trace elements in *P. perfoliatum* is vital for revealing the therapeutic mechanism of this herb and guiding the development and quality control of drugs based on it, expanding the applications of this herb. However, only Mn has been researched so far, and the correlation between the inorganic elements and bioactive compounds in *P. perfoliatum* has not been studied [8,9].

Ultrasonic-assisted extraction (UAE) is a popular and efficient extraction method for the preparation of Chinese herbal medicines [10]. Compounds with biological activity can be extracted faster and more completely by means of UAE compared with traditional extraction methods. High-performance liquid chromatography (HPLC), which usually follows UAE, is an essential method in the quality control of medicinal products [11].

Currently, inductively coupled plasma–atomic emission spectroscopy (ICP-AES), inductively coupled plasma–mass spectroscopy (ICP-MS), and inductively coupled plasma–optical emission spectroscopy (ICP-OES) are the three most common analysis techniques for inorganic elements [12]. Among these, ICP-AES is the most commonly used technique for the qualitative and quantitative analysis of inorganic elements in soil, biological samples, and cosmetic products. It is also widely used for determining the amount of inorganic elements in Chinese herbal medicines, ensuring that they are within acceptable levels [12]. ICP-AES has a low detection limit, high sensitivity, and high precision. Therefore, it was selected in this study as a method for determining the levels of inorganic elements in *P. perfoliatum* samples with different geographical origins.

*P. perfoliatum* has been identified to contain primary bioactive constituents such as flavonoids, quinones, and terpenoids, along with inorganic elements including Ca, Mg, Fe, Cu, Zn, and Mn, as well as trace heavy metals such as Pb, Hg, As, and Cd [13,14]. The flavonoid content and inorganic element profiles exhibit significant regional variations. For instance, samples from Cenxi demonstrated the highest flavonoid concentration at 2.19%, whereas those from Hengyang displayed the lowest level at 0.71% [15]. The comparative analysis of inorganic elements revealed that Mg and Mn levels in specimens from Sichuan Province surpassed those from Jiangxi Province, while Jiangxi-derived samples exhibited higher Fe contents than their Sichuan counterparts [16]. Another study predicted the suitable distribution of *P. perfoliatum*. The results show that the dominant environmental factors for *P. perfoliatum* are the precipitation in the wettest month, the annual average precipitation, and the precipitation in June. Highly suitable areas for *P. perfoliatum* include Fujian, Zhejiang, Jiangxi, Chongqing, Guangdong, and other regions [17]. However, the interconnections among its bioactive compounds, inorganic elements, and environmental factors are not yet clear.

This study selected 15 samples from 13 provinces with high yields and accessibility, namely Fujian, Guangxi, Anhui, Zhejiang, Jiangxi, Guizhou, Guangdong, Henan, Yunnan, Sichuan, Hunan, and Hebei. The aim of this study was to establish an HPLC method for the detection of multiple components in *P. perfoliatum* under a specific condition, to reveal the levels of bioactive compounds and inorganic elements in *P. perfoliatum* samples of different geographical origins, establish their associations, and provide a foundation for further investigation into *P. perfoliatum*.

## 2. Results and Discussion

The different geographical origins of the 15 *P. perfoliatum* used in this study are described in the Table 1. These *P. perfoliatum* samples are collectively referred to as *P. perfoliatum-x.*

### 2.1. HPLC Analysis of Bioactive Compounds in P. perfoliatum

Based on the criteria that all five major components could be detected under the same conditions, with a good resolution and baseline, water was selected as the extraction solvent, with extraction conducted at room temperature using ultrasonic waves for 40 min. Due to the main bioactive compounds in *P. perfoliatum* being flavonoids and phenolic acids, wavelengths of 285 nm and 320 nm were employed for detecting key *P. perfoliatum* (KP) bioactive compounds (caffeic acid, gallic acid, quercetin, isorhamnetin, and hyperoside) in *P. perfoliatum-x* [18]. When the detection wavelength was 285 nm, peak elution was concentrated in the first 20 min of the HPLC run, with poorer peak separation and a less smooth baseline. On the other hand, when the detection wavelength was 320 nm, better peak separation and a smoother baseline were observed. Therefore, 320 nm was selected as the detection wavelength for the subsequent HPLC analysis of standard compounds and *P. perfoliatum-x*.

#### 2.1.1. Method Validation

The *R*^2^ values of the standard curves for 5 KP bioactive compounds (caffeic acid, gallic acid, hyperoside, quercetin, and isorhamnetin; Table 2) were in the range of 0.9990 to 1.0000, indicating that the standard curves exhibited good linearity and they were suitable for use in determining the levels of relevant KP bioactive compounds and inorganic elements in *P. perfoliatum-x*. The average recovery rates were 87–105% (Table S1). The RSDs of the measurements for evaluating the precision, repeatability, and stability of the developed HPLC method were below 3%, indicating the validity of the method (Tables S2–S4).

#### 2.1.2. Quantitative Analysis of KP Bioactive Compounds

**Figure 1:**
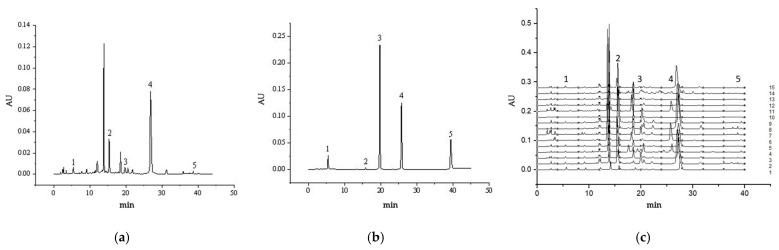
HPLC chromatograms of (**a**) the mixed standard solution in 320 nm (1: gallic acid, 2: caffeic acid, 3: hyperoside, 4: quercetin, and 5: isorhamnetin), (**b**) PPL-1, (**c**) *P. perfoliatum*-1, and *P. perfoliatum-x*. (Geographical origins of *P. perfoliatum*-1 to *P. perfoliatum*-15 are shown in Table 1).

The HPLC chromatogram of *P. perfoliatum*-1, which was selected as a representative of *P. perfoliatum-x*, was compared with that of the mixed standard solution (Figure 1). The HPLC detection wavelength was 320 nm. The RSDs of the retention times were less than 3%, indicating that the *P. perfoliatum* sample contained gallic acid, caffeic acid, hyperoside, quercetin, and isorhamnetin.

The standard curves for caffeic acid, gallic acid, hyperoside, quercetin, and isorhamnetin exhibited good linearity (*R*^2^ = 0.9987–0.9996, Table 2). These curves were used to obtain the concentrations by substituting the peak area into the equation. Subsequently, these concentrations were utilized in Equation (1) [19] to calculate the levels of KP bioactive compounds in *P. perfoliatum-x* (Table 3).

Quercetin emerged as the most prominent bioactive compound within the KP category in *P. perfoliatum-x* (as detailed in Table 3), closely followed by caffeic acid, hyperoside, gallic acid, and isorhamnetin in descending order. The concentration of quercetin in *P. perfoliatum-x* varied significantly, spanning from 15.67 μg/g to 50.19 μg/g. However, it is noteworthy that quercetin was absent in *P. perfoliatum*-4, *P. perfoliatum*-9, and *P. perfoliatum*-10, which correspond to samples sourced from Taizhou, Yizhou, and Meizhou, respectively.

On the other hand, the levels of gallic acid in *P. perfoliatum-x* were relatively modest, ranging between 0.49 μg/g and 2.21 μg/g. Intriguingly, this bioactive compound was undetectable in *P. perfoliatum*-6 and *P. perfoliatum*-11, originating from Guizhou and Fujian, respectively. All examined *P. perfoliatum-x* samples contained caffeic acid, with concentrations fluctuating between 4.43 μg/g and 28.35 μg/g.

Regarding hyperoside, its levels in *P. perfoliatum-x* ranged from 0 μg/g to 18.31 μg/g, with a notable absence in *P. perfoliatum*-12, the sample hailing from Kunming. Similarly, the levels of isorhamnetin in *P. perfoliatum-x* varied from 0 μg/g to 1.43 μg/g, with *P. perfoliatum*-10 being the only sample in which it was not detected.

The coefficients of variation (CVs) for all five components were above 30%, with quercetin and hyperoside exhibiting particularly high CVs of up to 50%. These findings highlight the variable presence and concentration of these bioactive compounds across different *P. perfoliatum* samples, suggesting potential geographical or environmental influences on their biosynthesis and accumulation.

Quercetin, isorhamnetin, and gallic acid exert anti-inflammatory effects by scavenging free radicals and inhibiting the NF-κB pathway [20,21], thereby supporting the “heat-clearing and detoxifying” efficacy of *P. perfoliatum*. Caffeic acid, quercetin, and gallic acid demonstrate “antiviral” efficacy by inhibiting viral neuraminidase, DNA gyrase, and bacterial biofilm formation [22,23,24]. Hyperoside regulates the glomerular filtration rate and aquaporin expression, supporting the “diuretic and anti-edema” effect [25].

#### 2.1.3. Results and Analysis of PCA and HCA of KP Bioactive Compounds

The effects of geographical origin on the levels of KP bioactive compounds in *P. perfoliatum-x* were evaluated by performing PCA and HCA using SPSS 26.0.

**Figure 2:**
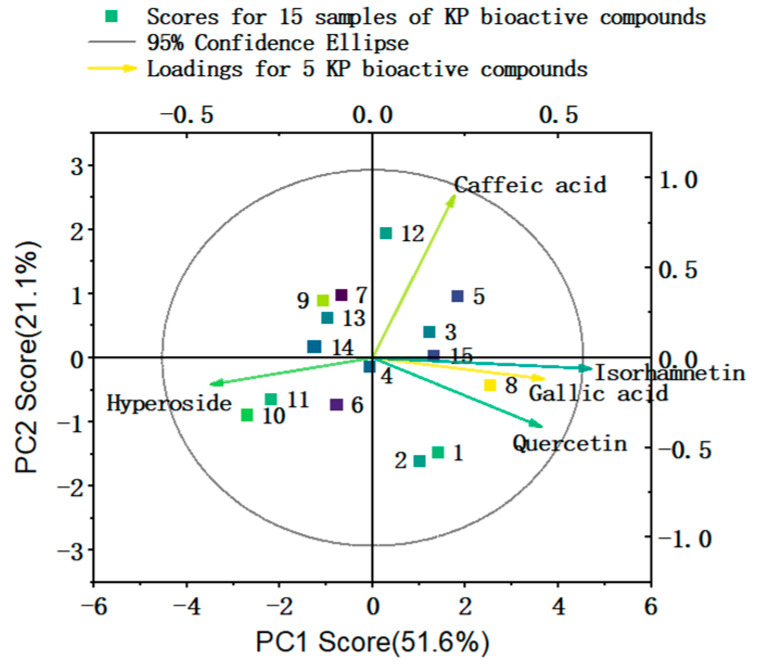
The Score and Loading plot of KP bioactive components.

The eigenvalues of the first (PC1) and second (PC2) principal components were greater than 1 (Table S1), and the cumulative variance contribution rate was greater than 70%, indicating that the two principal components were able to explain most of the variance in the levels of KP bioactive compounds in *P. perfoliatum-x*. The correlations of PC1 and PC2 with the levels of KP bioactive compounds in *P. perfoliatum-x* were analyzed by constructing a component score coefficient matrix (Table S2) [26]. PC1 was mainly correlated with the levels of isorhamnetin, hyperoside, quercetin, and gallic acid in *P. perfoliatum-x*, while PC2 was mainly correlated with the levels of caffeic acid in the *P. perfoliatum* samples. The levels of hyperoside in *P. perfoliatum-x* were negatively correlated with PC1 and PC2, while the levels of caffeic acid in the *P. perfoliatum* samples were positively correlated with PC2 (Table S2). Compared with PC2, PC1 explained more of the total variance. From the Score and Loading plot of KP bioactive components, it is apparent that isorhamnetin, gallic acid, and quercetin exhibit relatively strong correlations. Despite having the lowest content in *P. perfoliatum-x*, isorhamnetin makes a notably high contribution to the principal component. The similarities observed among the samples are consistent throughout. The first principal component (PC1) accounts for 51.6% of the variation, while the second principal component (PC2) explains 21.1% (Figure 2).

**Figure 3:**
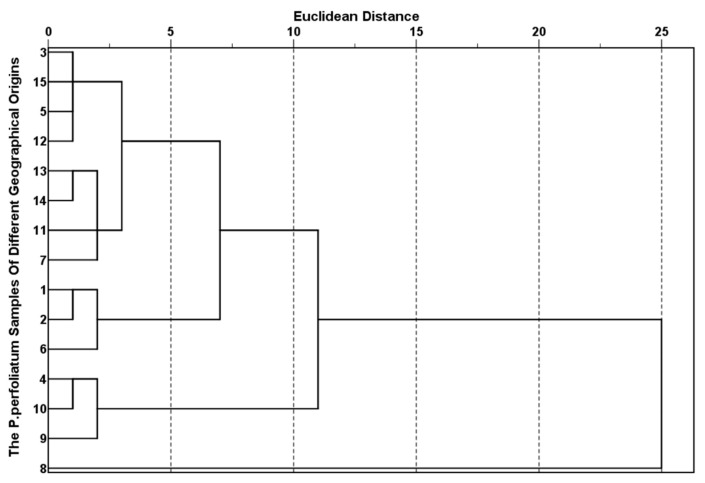
Dendrogram of HCA of KP bioactive compounds in *P. perfoliatum-x*.

The results of HCA clustered *P. perfoliatum-x* into four categories (Figure 3) on the basis of the levels of KP bioactive compounds in the *P. perfoliatum* samples from different geographical origins, with the distance between categories being five. 

*P. perfoliatum*-8 was clustered into category 1; *P. perfoliatum*-1, *P. perfoliatum*-2, and *P. perfoliatum*-6 were clustered into category 2; *P. perfoliatum*-4, *P. perfoliatum*-9, and *P. perfoliatum*-10 were clustered into category 3; the other *P. perfoliatum* samples were clustered into category 4 [27], demonstrating that there were significant differences in the levels of KP bioactive compounds between *P. perfoliatum*-8 and the other *P. perfoliatum* samples. *P. perfoliatum*-8 was collected from Luoyang (Henan Province). This region exhibits a temperate monsoon climate (Δ15.6 °C seasonal temperature variation, 650 mm concentrated rainfall), and it is surrounded by mountains. This uniqueness is due to the synergistic regulation of flavonoid synthesis pathways, especially for quercetin, by the mean annual precipitation concentration. *P. perfoliatum*-1, *P. perfoliatum*-2, and *P. perfoliatum*-6 are classified into one category. All three regions are located in the southern part of China, with Wuyishan having an elevated and steep terrain, Hechi having more mountains than arable land and containing a high distribution of karst [28], and Guiyang located in the eastern part of the Yunnan–Guizhou Plateau, with a relatively undulating terrain. All three regions have a relatively complex topography and landforms, and *P. perfoliatum* from these areas is characterized by its high quercetin content.

The annual average temperature, the annual average maximum temperature, the average minimum temperature, the average rainfall, and the annual average sunshine duration of the production origins 4, 9, and 10 are all very similar. Zhejiang, Guangxi, and Guangdong are all coastal provinces. Moreover, the geographical locations of the collected samples are all north of the Tropic of Cancer. Among them, *P. perfoliatum*-4 and *P. perfoliatum*-10 have a subtropical monsoon climate, so these three origins are grouped into one category. *P. perfoliatum*-9 was collected from Yizhou, which has a semi-mountainous and semi-hilly terrain and a wealth of precipitation, rivers, and water resources [29,30]. It is also noted that the contents of quercetin in these three samples are extremely low.

PCA and HCA jointly revealed the differences in bioactive substances among 15 origins and their environmental driving factors from two perspectives: the variation direction and contribution degree of chemical composition and geographical classification. The key components extracted via PCA (PC1 flavonoids, PC2 caffeic acid) highly matched the geographical clustering of HCA, indicating that the differences in chemical composition have a clear environmental driving basis. The results of the two methods are highly synergistic.

### 2.2. Analysis of Inorganic Elements

#### 2.2.1. Quantitative Analysis of Inorganic Elements

The linear regression equations and *R*^2^ values of the standard curves for the 13 inorganic elements are shown in Table 4. The *R*^2^ values were greater than 0.9990, indicating that the standard curves exhibited good linearity [31].

The levels of the 13 inorganic elements in *P. perfoliatum-x* are shown in Table 5.

The most abundant inorganic element in *P. perfoliatum-x* was Ca (712.47 µg/g), followed by Mg (166.73 µg/g), Al 58.95 µg/g), and Fe 54.76 µg/g). On the other hand, the least abundant inorganic element in *P. perfoliatum-x* was Ni (1.158 µg/g), followed by Pb (2.02 µg/g) and Cu (2.61 µg/g). The levels of the remaining inorganic elements were moderate [32].

The presence of Ca in plants is related to the formation of calcium pectate, calmodulin, and calcium magnesium phytate, which allows Ca to help stabilize the cell membrane and cell wall. In addition, Ca participates in signal transmission, osmosis regulation, and enzymatic reactions [33].

Mg influences plant photosynthesis because it is one of the main components of chlorophyll. It can stimulate the activities of ATP kinase, glucose kinase, fructose kinase, and phosphoglucomutase, and therefore, it plays important roles in carbohydrate metabolism, DNA synthesis, maintenance of immune cell function, and fatty acid synthesis [34]. Moreover, Mg is the second most prevalent electrolyte and the fourth most abundant metal in the human body [35]. It can help to reduce blood pressure, increase Ca absorption, and protect against anemia, and Mg supplementation has been used as a supporting treatment for cancer [36].

The Fe levels in *P. perfoliatum-x* ranged from 12.5 µg/g to 190 µg/g, and the Zn levels in the *P. perfoliatum* samples ranged from 2.05 µg/g to 10.80 µg/g. Zn and Fe are essential trace elements in the human body, and Fe is an important component of hemoglobin [37,38]. The latter also serves as a cofactor in cytochrome P450 enzymes, playing a crucial role in the metabolism of various trace elements and contributing to the enhancement of immune function and anemia prevention. Zn is an antioxidant enzyme (SOD) cofactor, frequently utilized as an anticancer adjuvant because it has a cancer-preventative impact [37,38]. Zn deficiency can cause a number of illnesses, but excessive Zn intake can lead to poisoning, acute renal failure, or even sudden death. An excessive iron intake increases the burden on the liver, and it can cause liver cirrhosis, atherosclerosis, or tumors [39,40] (Table 5).

The Cu levels in *P. perfoliatum-x* ranged from 0.454 µg/g to 4.920 µg/g, within the 20 mg/kg limit specified by the Chinese Pharmacopeia [41]. Although Cu is a micronutrient for plants, it is moderately phytotoxic at high doses [42]. Cu is an anti-inflammatory enzyme (superoxide dismutase) cofactor that inhibits inflammatory factors such as IL-6, but excessive Cu consumption is harmful to humans [42].

Pb is not an essential element in plants and is usually harmful to them; its levels in *P. perfoliatum-x* ranged from 1.030 µg/g (in *P. perfoliatum*-2) to 3.630 µg/g (in *P. perfoliatum*-13), being lower than the 10 µg/g limit recommended by the Chinese Pharmacopeia for medicinal plants [43]. Pb is an environmental contaminant that can cause significant harm to human health because it can accumulate in the bone marrow and skeleton, negatively affecting brain function and development and increasing the risks of cognitive and behavioral problems [44].

The As levels in *P. perfoliatum-x* exceeded the 2 µg/g limit specified by the Chinese Pharmacopeia, indicating the bioaccumulation of As in *P. perfoliatum*. As is a heavy metal with significant biotoxicity [41]. It is carcinogenic, and long-term intake may lead to skin cancer and other diseases. Therefore, the subtle toxic effects of *P. perfoliatum* could be attributed to its excessive As level.

Cr, Ba, and Ni were present in *P. perfoliatum-x* at low concentrations (less than 3 µg/g). These elements are heavy metals and can pose a threat to human health because of their toxicity and bioaccumulation tendency [1]. The excessive intake of barium (Ba) may result in muscle paralysis and other associated symptoms, while excessive exposure to nickel (Ni) has been reported to induce allergic reactions in certain individuals. Hexavalent chromium is classified as carcinogenic, whereas trivalent chromium is recognized as an essential trace element for the human body. Nevertheless, excessive intake of trivalent chromium may still pose potential health risks. While there is no limit for Ba, Ni, and Cr individually, the combined levels of these heavy metals in *P. perfoliatum-x* exceeded the 20 mg/kg limit suggested by Li et al. [45].

The Mn levels in *P. perfoliatum-x* ranged from 0 µg/g to 51.3 µg/g. Mn was not detected in *P. perfoliatum*-3, *P. perfoliatum*-11, *P. perfoliatum*-12, *P. perfoliatum*-13, *P. perfoliatum*-14, or *P. perfoliatum*-15. Mn is an essential element for the human body because it is present in all types of tissue. It serves as an antioxidant, and it plays crucial roles in immune function, blood sugar regulation, cellular reproduction, digestion, energy production, and bone formation [46]. Moreover, it exhibits other beneficial properties, such as having anticancer [47] and antibacterial [48] properties.

Se is an essential inorganic element for the survival of humans and animals. It serves as a cellular antioxidant, and Se supplementation has been used as an adjuvant treatment for cancer and cardiovascular disease. Moreover, Se possesses antiviral properties, and thus, the antiviral properties of *P. perfoliatum* can be attributed to its Se content [49]. Se-containing Chinese medicinal formulations can prevent tumor growth and exhibit antimicrobial actions against pathogenic microorganisms [50].

The coefficients of variation (CVs) for Fe, Mn, Ni, Al, and Zn exceed 50%, indicating that environmental changes have a significant impact on their concentrations, with Mn showing a particularly high CV of 130%. In contrast, the CVs for As, Ca, Cr, Mg, and Se fall within the 20–30% range, suggesting that *P. perfoliatum* has relatively stable absorption and transport mechanisms for these elements.

#### 2.2.2. Results of HCA and PCA of Inorganic Elements

The effects of geographical origin on the levels of inorganic elements in *P. perfoliatum* were evaluated by conducting PCA and HCA. The eigenvalue of each of the first four principal components (PC1, PC2, PC3, and PC4) was greater than one (Table S3), and the cumulative variance contribution rate of the four principal components reached 80.71%, indicating that the principal components could explain most of the variance in the levels of inorganic elements in *P. perfoliatum-x* [51].

**Figure 4:**
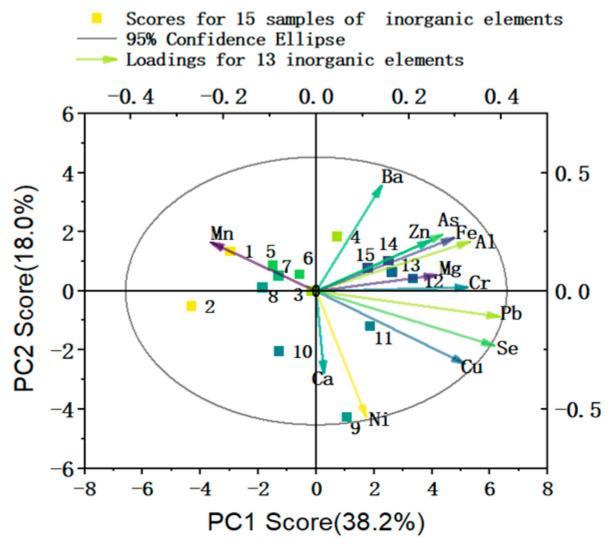
The Score and Loading plot of elements.

The correlations of the four principal components with the levels of different inorganic elements in *P. perfoliatum-x* were evaluated by constructing a component score coefficient matrix (Figure 4, Table S4).

The four principal components (PC1, PC2, PC3, and PC4) were correlated with Pb, Se, Al, Cr, Cu, Fe, Zn, Ni, Ba, Ca, Mg, As, and Mn levels in *P. perfoliatum-x*. PC1 was positively correlated with Pb, Se, Al, Cr, Cu, Fe, and Zn levels in *P. perfoliatum,* and PC3 was positively correlated with Mg and As levels in *P. perfoliatum*, while PC4 was positively correlated with Mn levels in *P. perfoliatum* samples [52]. PC2 was positively correlated with Ba levels in *P. perfoliatum* but negatively correlated with Ni and Ca levels. The correlations among Zn, As, Fe, Al, Mg, Cr, Pb, Se, and Cu are relatively high, among which Pb, Se, Cu, and Al have a greater contribution to the principal component. In addition, the similarities among the samples are consistent.

**Figure 5:**
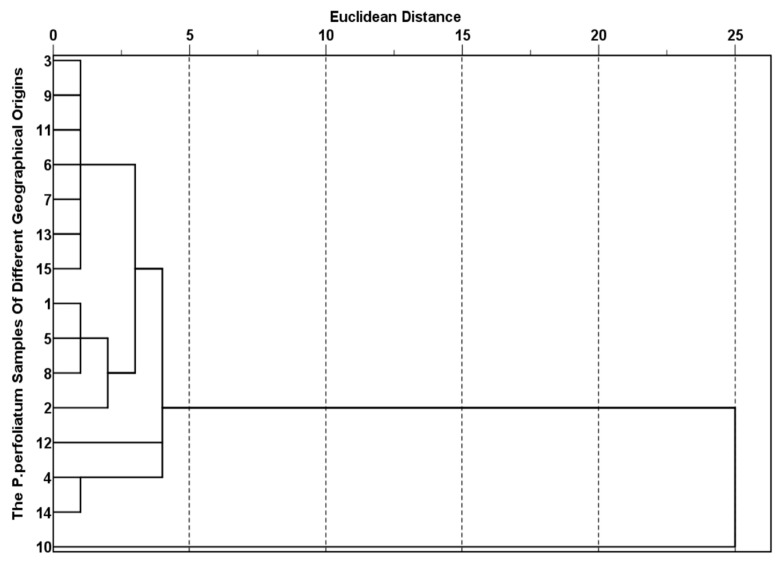
Dendrogram of HCA of inorganic elements in *P. perfoliatum-x*.

The results of HCA clustered *P. perfoliatum-x* into two categories on the basis of the levels of inorganic elements in the *P. perfoliatum* samples of different geographical origins (Figure 5), with *P. perfoliatum*-10 and the remaining *P. perfoliatum* samples being clustered into separate categories. The distance between the categories was five. Meizhou is located south of the Five Ridges (the Wuling Mountains), with approximately 85% of the area being hilly and mountainous regions that are below 500 m above sea level. It is situated in a monsoon-affected subtropical climate zone with sufficient sunlight and rainfall, and it exhibits a multilevel non-zonal climate due to its complex terrain [53]. The content of Ca, which is negatively correlated with PC2, is significantly higher in *P. perfoliatum*-10 compared with samples from other origins, possibly because of the local limestone geology, so it is classified as a separate category.

The contents of Fe and Al, which are correlated with PC1, are notably higher in samples 4, 12, and 14 compared with samples from other origins, likely owing to acidic soil, and thus they are grouped into one category. However, sample 12 also has a relatively high calcium content and is classified in a separate category. Sample 12 is from Yunnan, which is a mountainous plateau with different soil qualities and a monsoon-affected subtropical climate [54]. *P. perfoliatum*-4 and *P. perfoliatum*-14, which were obtained from Taizhou and Baoding, respectively, were grouped together. Both Taizhou and Baoding are in industrial regions [55].

*P. perfoliatum*-1, *P. perfoliatum*-5, *P. perfoliatum*-8, and *P. perfoliatum*-2, which were obtained from Wuyishan, Ganzhou, Luoyang, and Hechi, respectively, were grouped together. Wuyishan and Ganzhou are situated in neighboring provinces; Luoyang and Wuyishan belong to mountainous areas, while Hechi and Wuyishan are close to railways [56]. The samples all exhibit relatively low Cu and Ca contents, with sample 2, in particular, having a Ca content lower than the other three samples and thus being classified in a separate category. The samples all exhibit a relatively high Ca content, as well as medium–high levels of Al, Fe, and Cu.

*P. perfoliatum*-3, *P. perfoliatum*-9, *P. perfoliatum*-11, *P. perfoliatum*-6, *P. perfoliatum*-7, *P. perfoliatum*-13, and *P. perfoliatum*-15 were grouped together. These *P. perfoliatum* samples were collected from regions in southern China (Anhui, Guangxi, Fujian, Guizhou, Guangdong, Hunan, and Sichuan, respectively). Guangxi, Guangdong, and Fujian are coastal areas in southern China. Sichuan and Anhui are located adjacent to the Yangtze River, while Guizhou and Hunan are situated adjacent to the Yunnan–Guizhou Plateau. Except for Guizhou, these regions are rich in water resources.

### 2.3. Correlation Analysis

The correlations between the levels of inorganic elements and levels of KP bioactive compounds in *P. perfoliatum-x* were analyzed using SPPS 26.0, and the results are shown in Table 6.

Isorhamnetin demonstrated a significant negative correlation with Ca content, suggesting that Ca may influence physiological processes associated with isorhamnetin synthesis [57]. Similarly, quercetin exhibited a pronounced correlation with Al and Cr concentrations, implying that reduced Al and Cr levels could induce flavonoid biosynthesis. These findings align with the mechanistic insights reported by GuoXuan Liu et al. [58], who demonstrated that GmSTOP1-3 enhances aluminum tolerance in soybean by regulating flavonoid synthesis to reduce reactive oxygen species (ROS) accumulation [59]. Notably, Mn displayed positive correlations with the contents of the five KP bioactive compounds. Both Mn and these bioactive compounds are recognized for their anticancer properties [60,61,62,63,64,65]. Furthermore, a negative correlation was observed between Ca levels and the concentrations of isorhamnetin and quercetin, which aligns with prior findings on flavonoids’ inhibitory effects on Ca^2+^ accumulation in lens tissues [66].

The concentrations of Mg and Zn demonstrated significant positive correlations with caffeic acid, hyperoside, and isorhamnetin. This observation suggests that Mg and Zn may directly or indirectly modulate the activity of the antioxidant enzyme system through multiple biochemical pathways, thereby promoting the biosynthesis of these phenolic compounds. These findings align with prior multi-elemental analyses conducted by Tkacz et al., who reported positive correlations between Mg/Zn levels and isorhamnetin/caffeic acid contents in sea buckthorn berry tissues, branches, and leaves [34,67]. Notably, selenium (Se) concentrations exhibited a positive association with caffeic acid and hyperoside levels. Such correlation may reflect synergistic interactions between Se’s pharmacological properties—including immunomodulatory, anti-aging, and antitumor effects—and the documented antioxidant, antiviral, and immune-potentiating activities of caffeic acid and hyperoside [68]. This mechanistic parallelism is further supported by Jingzhou Dong et al.’s [69] investigation into bioactive component distribution in selenium-enriched *Cordyceps militaris* fruit bodies, which revealed analogous co-accumulation patterns of selenized metabolites and phenolic acids.

Quercetin and isorhamnetin demonstrated a negative correlation with heavy metal elements (Cr, Cu, and Pb), indicating that these flavonoids can inhibit the occurrence of heavy metal elements, suggesting a potential inhibitory effect of these flavonoids on heavy metal bioaccumulation. This phytochemical–metal interaction may contribute to mitigating heavy metal toxicity in biological systems, thereby preserving the phytotherapeutic integrity of *P. perfoliatum* and enhancing its pharmacological safety profile. These observations corroborate findings by Kostic et al., who identified inverse relationships between flavonoid content and toxic metal concentrations in Balkan medicinal plants [70], as well as Boyarskikh et al.’s altitudinal gradient analysis of *Lonicera caerulea* subsp. *altaica*, which revealed metal–flavonoid antagonism across plant organs. Collectively, these data establish a significant interdependence between inorganic element profiles and bioactive compound concentrations in *P. perfoliatum*. Notably, the elemental composition of medicinal plants is modulated by multifaceted environmental and agronomic variables, including taxonomic specificity, edaphic characteristics (e.g., pH, organic matter content, etc.), microclimatic conditions, and cultivation management strategies [71].

This study conducted Pearson correlation coefficient (α = 0.05) analysis using SPSS 26.0 (IBM Corp., Chicago, IL, USA) software to investigate the interrelationships among KP bioactive compounds, inorganic elements, and climatic parameters in *P. perfoliatum*. The results are presented in Table 7 and Table 8.

As illustrated in Table 7, the impact and orientation of diverse environmental parameters on the composition of bioactive compounds in *P. perfoliatum* differ.

Temperature emerged as a pivotal factor influencing the accumulation of flavonoids, exhibiting a bidirectional response pattern. Specifically, the contents of quercetin and isorhamnetin demonstrate negative correlations with annual mean temperature, as well as its minimum and maximum values. The correlation coefficients between quercetin and these three temperature indicators (annual mean temperature, the minimum value of annual mean temperature, and the maximum value of annual mean temperature) did not differ significantly. In contrast, the absolute value of the correlation coefficient of isorhamnetin with the maximum average temperature was 0.363, which was significantly higher than those of the other two correlations (with annual mean temperature and the minimum value of annual mean temperature), indicating that a high temperature is not conducive to the accumulation of isorhamnetin. This finding is consistent with the observations of Chen Lei et al., who also reported a decrease in the content of ginkgo flavonoids under high/normal temperature and drought stress conditions [72]. Conversely, hyperoside, which is formed by the linkage of a β-glycosidic bond between the 3-hydroxy group of quercetin and galactopyranose, exhibits a positive correlation with temperature. In particular, its correlation coefficient with the annual average minimum temperature reached 0.411. Moreover, hyperoside shows a negative correlation with altitude, suggesting that low-temperature stress is beneficial for its accumulation. This aligns with the research conducted by Wang Guibin et al., which revealed that the content of soluble sugar in Ginkgo leaves increases under lower temperatures (below 15 °C) and humid conditions, thereby facilitating the biosynthesis of flavonoid compounds [73].

Furthermore, hyperoside differs from quercetin in that the values of its correlation coefficients with annual average precipitation are greater than 0.3, indicating that precipitation is conducive to its accumulation. This is consistent with the findings of Jiang-Fei Meng et al., who observed that the content of most phenolic compounds in grape samples was lower under rain-shelter cultivation compared with open-field cultivation [74].

The influence of environmental factors on the accumulation of flavonoid glycosides and flavonoid aglycones in *Polygonatum odoratum* varies significantly. Hyperoside is the most susceptible to environmental factors, and its underlying mechanism warrants further investigation.

Gallic acid can be biosynthesized in plants through the phenylpropanoid, shikimic acid, and tannin degradation pathways. As presented in Table 7, the environmental factors with absolute correlation coefficient values exceeding 0.2 are altitude and mean annual precipitation. The negative correlation observed between gallic acid content and altitude is consistent with the findings of Xiaobo Zhang et al., who reported that *Polygonum capitatum* exhibits a higher gallic acid content in lower-altitude, shady-slope, and smaller-slope areas [75]. Conversely, the positive correlation between gallic acid content and precipitation aligns with the research conducted by Ren Min, which demonstrated that water stress can significantly enhance gallic acid levels in *Lespedeza davurica* [76].

The biosynthesis of caffeic acid is primarily dependent on the activities of phenylalanine ammonia-lyase and cinnamate 4-hydroxylase within the phenylpropanoid metabolic pathway [77]. Notably, the absolute values of the correlation coefficients between caffeic acid and all other environmental factors were below 0.3, indicating relatively weak correlations. This suggests that environmental factors exert a minimal influence on caffeic acid content, a conclusion that is consistent with the results of the principal component analysis (PCA). Furthermore, caffeic acid exhibits a negative correlation with altitude, a finding that corroborates the research reported by Hasan Oruç et al., who observed that propolis collected from low-altitude areas contains higher levels of caffeic acid compared with that from high-altitude regions [78].

As shown in Table 8, a complex interplay exists between inorganic element contents and climatic factors in *P. perfoliatum*. These correlations are mediated by multiple factors, including the geochemical attributes of elements, the mechanisms by which plants absorb and transport these elements, and the intricate interactions among various climatic factors.

I. Dominant Temperature Response Patterns

A predominant negative correlation was observed between temperature metrics (annual mean, maximum, and minimum) and concentrations of Al, As, Ba, Cr, Fe, Mg, Pb, Se, and Zn. Notably, As and Mg exhibited the strongest temperature sensitivity, particularly with respect to maximum temperature. Sardans et al. [79] demonstrated a 19% increase in Mg accumulation in soil exchange complexes under warming scenarios—a phenomenon potentially linked to reduced phloem-mediated Mg translocation. Concurrently, Zhai et al. [80] reported decreased As bioavailability in rice shoots under experimental warming, consistent with our findings of temperature-dependent As sequestration in *P. perfoliatum* [81].

Ca, Mn, and Ni exhibited positive correlations with thermal parameters, though statistical significance thresholds were not universally met (*p* > 0.05). Specifically, the Ca response aligns with Sardans et al. [79], who documented a 42% increase in foliar Ca concentrations in *Erica multiflora* under experimental warming—potentially attributable to temperature-enhanced soil cation exchange capacity.

Cu displayed temperature-indifferent accumulation patterns, consistent with Xu et al. [82], demonstrating temperature-independent Cu uptake dynamics [83].

II. Divergent Impacts of Precipitation and Elevation

Predominantly Negative Correlation with Precipitation: Al, As, Cr, Cu, Fe, Mg, Se, and Zn exhibit negative correlations with annual mean precipitation.

Mn as an Exception: Mn stands out as the sole element displaying a significant positive correlation with precipitation, consistent with Jian Feng et al.’s research indicating that increased precipitation promotes Mn release [80].

Positive Correlation with Elevation: With the exception of Ca, Mn, and Ni, all other elements show a positive correlation with elevation. Notably, Fe demonstrated a significant positive correlation, indicating enhanced Fe mobility and availability in high-altitude soils. Al has a correlation coefficient exceeding 0.3, suggesting substantial variations in its concentration with altitude. In the oxygen-deficient environment at high altitudes, the respiratory processes of plants are inhibited, necessitating an enhanced iron-dependent cytochrome pathway to sustain energy supply. This finding aligns with the research conducted by Ma Jianhua et al. [84].

III. Bidirectional Regulation by Sunshine Duration

Positive Correlation Group: Al, Cr, and Fe exhibit a significant positive correlation with annual mean sunshine duration. This correlation may stem from intense sunlight-promoting soil oxidation processes, such as Fe^3+^ precipitation or the utilization of metal ions in plant photoprotection mechanisms [85].

Negative Correlation Group: Ca, Mg, Mn, and Zn display a negative correlation with sunshine duration. This trend may be attributed to prolonged sunshine duration accelerating plant growth, thereby depleting nutrient elements, or to reduced absorption efficiency resulting from light inhibition. This is consistent with a study by Zha Ling Yan et al. on the effect of continuous light on mineral element absorption in lettuce. Compared with conventional light, continuous light for 30 days significantly reduced the Ca, Mg, Mn, and Zn content of lettuce [86].

Ba and Ni exhibit climate-insensitive biogeochemical stability, showing no statistically significant associations with mean annual rainfall, elevation, or annual sunshine duration.

The complex correlations between inorganic element contents and climatic factors in *P. perfoliatum* hold considerable promise for application in ecosystem management, agricultural and forestry practices, climate change research, environmental policy formulation, and biotechnological applications.

## 3. Materials and Methods

### 3.1. Materials

In compliance with regulations and ecological protection, and with permission for the collection, the aerial parts of *P. perfoliatum* samples from 15 different geographical origins (*P. perfoliatum*-1 to *P. perfoliatum*-15, Table 1) were collected in September 2022 (in the summer when *P. perfoliatum* flowers bloomed) and identified by Professor Wu Yanfang (School of Life Science and Technology, Henan Institute of Science and Technology). These *P. perfoliatum* samples were collectively referred to as *P. perfoliatum-x*. The standard compounds for caffeic acid, gallic acid, quercetin, isorhamnetin, and hyperoside were purchased from Shanghai Aladdin Bio-Chem Technology (Shanghai, China). HPLC-grade methanol was obtained from Xilong Scientific Co., Ltd. (Shantou, China). HPLC-grade acetonitrile and HPLC-grade acetic acid were supplied by Tianjin Kemiou Chemical Reagent Co., Ltd. (Tianjin, China). A multi-element ICP standard solution (1000 ppm) was provided by the China National Center for the Quality Supervision and Testing of Iron and Steel (Beijing, China). All other reagents and solvents were of analytical grade.

### 3.2. Methods

#### 3.2.1. HPLC Analysis

##### 3.2.1.1. HPLC Sample Preparation

Sample preparation was conducted based on the methods reported by Dastan et al. [15] and Ke et al. [16], with some modifications. *P. perfoliatum* was dried in the shade, powdered, and passed through a 50-mesh sieve. The *P. perfoliatum* powder (5 g) was added to a 100 mL round-bottomed flask, and it was extracted using a Ymnl-2008DE intelligent temperature-controlled dual-frequency ultrasonic extractor (Nanjing Emmanuel Instruments Co., Ltd., Nanjing, China) with distilled water (50 mL) for 40 min at 25 °C, using a power setting of 600 W. The extract was filtered, and the filtrate was centrifuged at 4000 rpm for 10 min. The supernatant was evaporated to dryness and then dissolved with 10 mL of HPLC-grade methanol to obtain a sample for HPLC analysis, and the extraction efficiency generally ranged from 1% to 3%.

##### 3.2.1.2. Standard Solution Preparation and Method Validation

A mixed standard solution containing 0.414 μg/μL of isorhamnetin, 0.306 μg/μL of gallic acid, 0.386 μg/μL of quercetin, 0.560 μg/μL of hyperoside, and 0.360 μg/μL of caffeic acid in HPLC-grade methanol was prepared and diluted to form a series of working solutions for constructing standard curves by plotting the concentrations (μg/μL) of each standard compound against their corresponding peak areas, and linear regression equations were obtained for each compound. We conducted methodological investigations, including assessments of stability, precision, and repeatability, to ensure the feasibility of the detection method.

The linearity, precision, repeatability, stability, and accuracy of the developed HPLC method were validated according to the International Conference on Harmonization (ICH) guidelines for the validation of analytical methods [87]. Standard curves (peak area vs. concentration curves) for different standard compounds were constructed, and the linearity of the measurements was determined on the basis of the *R*^2^ values of the standard curves. The precision and repeatability of the method were evaluated by preparing six samples from *P. perfoliatum*-1 according to the sample preparation method described in Section 3.2.1.1 and analyzing the samples under the chromatographic conditions described in Section 3.2.1.2. On the other hand, the stability of the method was determined by varying the HPLC run time (0, 2, 4, 8, 12, 24, or 48 h) using recovery investigations of *P. perfoliatum*-2. Recovery investigations were conducted to evaluate the accuracy of the HPLC method. This was accomplished by adding a standard solution equivalent in chemical composition to that of *P. perfoliatum*-2, followed by extraction as outlined in Section 3.2.1.1. The process was repeated three times, and the average recovery rate of the spiked samples was calculated. The precision, repeatability, and stability of the method were evaluated based on six repetitions, using the relative standard deviations (RSDs) of the relevant measurements as the criteria for assessment.

##### 3.2.1.3. Sample Analysis

Sample analysis was conducted using an e2695 HPLC system (Waters Corporation, Milford, MA, USA) with a Symmetry C18 column (250 mm × 4.6 mm, 5 µm; Waters Corporation, USA). The experiment employed gradient elution with the following conditions: acetonitrile (A) and 0.5% acetic acid in water (B). The elution profile was as follows: 0–10 min, A: 5–18%, B: 95–82%; 10–30 min, A: 18–22%, B: 82–78%; 30–35 min, A: 22–35%, B: 78–65%; 35–40 min, A: 35–5%, B: 65–95%. The flow rate was 1 mL/min. The column temperature was 30 °C, and the injection volume was 10 μL. The eluent was monitored at 320 nm [6,88,89]. When the mobile phase contained acetonitrile and acetic acid aqueous solutions, the dissolution of water-based extracts in methanol helped to increase the efficiency of HPLC analysis, improve peak shape, reduce solvent effects, and ensure sample compatibility and safety during HPLC analysis. The level of each of the key *P. perfoliatum* bioactive compounds (KP bioactive compounds: isorhamnetin, gallic acid, quercetin, hyperoside, and caffeic acid) in each *P. perfoliatum-x* was calculated in the herbal drug using Equation (1) [19].

X = (C × V)/M,(1)

where X (μg/g) is the level of the bioactive compound in the sample, C (μg/μL) is the concentration of the bioactive compound in the sample (which was determined using the standard curve for the compound), V (μL) is the volume of the sample, and M (g) is the mass of the sample.

#### 3.2.2. ICP-AES Analysis

##### 3.2.2.1. ICP-AES Sample Preparation

The aerial parts (stems, leaves, and flowers) of *P. perfoliatum* (0.2 g) were placed in a digestion tank, and then nitric acid (8 mL) and hydrogen peroxide (1 mL) were added to the tank. The plant material was digested using a Multiwave PRO microwave digestion system (Anton Paar, Graz, Austria) by applying two-step temperature programming. During the first step, the temperature was increased to 120 °C within 8 min and then maintained for 5 min. During the second step, the temperature was raised to 190 °C within 8 min, maintained for 35 min, and then lowered to 55 °C. After the temperature programming was completed, the material was removed from the digestion tank.

##### 3.2.2.2. Quantitative Analysis

The 1000 ppm multi-element ICP standard solution was diluted with a 2% nitric acid solution to prepare multi-element working solutions with different concentrations (0.02 ppm, 0.1 ppm, 0.5 ppm, 1 ppm, and 5 ppm).

The working solutions were subjected to ICP-AES analysis [90,91] using an ICPE 9820 spectrometer (Shimadzu, Kyoto, Japan) to construct the standard curves for 13 inorganic elements (Al, As, Ba, Ca, Cr, Cu, Fe, Mg, Mn, Ni, Pb, Se, and Zn). Linear regression was applied to the standard curves, and the obtained regression coefficients (*R*^2^ values) were in the range of 0.9990 to 1.0000, indicating the good linearity of the standard curves. The operating parameters of the ICPE 9820 spectrometer were as follows: The incident power was 1.3 kW, the plasma gas flow rate was 15 L/min, the auxiliary gas flow rate was 0.2 L/min, and the atomizing gas flow rate was 0.8 L/min. Three replicates were prepared for each of *P. perfoliatum-x*.

#### 3.2.3. Statistical Analysis

All experiments were carried out in triplicate. The raw levels of inorganic elements and KP bioactive compounds in *P. perfoliatum-x* were subjected to principal component analysis (PCA) and hierarchical cluster analysis (HCA) by using SPSS 26.0. The method of cohesive hierarchical clustering was utilized, where each data point was treated as a separate cluster. Cluster analysis was then performed using the distance measure of Euclidean distance. The correlations between the levels of inorganic elements and levels of KP bioactive compounds in *P. perfoliatum-x* were determined using Pearson’s correlation analysis.

## 4. Conclusions

This study investigated the contents of inorganic elements and KP bioactive compounds in *P. perfoliatum* samples from 15 different geographical regions. Among the KP bioactive compounds in *P. perfoliatum*, quercetin exhibited the highest content, followed by caffeic acid, hyperoside, gallic acid, and isorhamnetin. Significant variations in the contents of these compounds were observed across geographical origins, highlighting distinct geographical characteristics, particularly isorhamnetin, hyperoside, quercetin, and gallic acid. Ca, Fe, Al, Mg, and Mn were the most abundant inorganic elements in the samples. *P. perfoliata* exhibits specific absorption characteristics for arsenic (As). The As content in samples with different origins consistently exceeds the limits specified in the Pharmacopeia, with a low coefficient of variation. The Pharmacopeia notes that *P. perfoliata* is slightly toxic, and this may be attributed to its As content. Additionally, its ability to accumulate As makes it applicable for polluted soil remediation. Mn and Fe showed pronounced sensitivity to environmental conditions, with their contents fluctuating significantly across regions.

HCA was used to classify samples into distinct clusters based on KP compounds and inorganic elements, which correlated with similarities in climate and altitude.

The correlations between climate and KP bioactive substances and inorganic elements were analyzed in detail. Significant correlations were identified between inorganic elements and KP compounds. For example, quercetin was negatively correlated with Al and Cr, while isorhamnetin showed a negative correlation with Ca. Mn was positively correlated with all five KP compounds, implying its potential role in promoting their biosynthesis.

Climatic factors such as temperature, precipitation, and sunlight duration significantly affected the accumulation of both inorganic elements and KP compounds. Temperature primarily influenced flavonoid synthesis, whereas precipitation impacted phenolic acid content. Heat stress significantly inhibits the biosynthetic pathways of quercetin and isorhamnetin while concurrently reducing the bio-enrichment capacity of As. Conversely, heavy precipitation environments enhance the dissolved-phase migration efficiency of Mn in soil, thereby facilitating the synthesis of hyperoside and establishing a synergistic Mn–hyperoside relationship.

In summary, this research not only reveals the spatial patterns and environmental drivers of bioactive compounds and inorganic elements in *P. perfoliatum* but also advances its potential applications in agriculture, ecology, and phytoremediation. These findings establish a theoretical foundation for the development, utilization, and traceability of medicinal plants.

## Figures and Tables

**Table 1:** Geographical origins of *P. perfoliatum*-1 to *P. perfoliatum*-15.

P. perfoliatum Samples	Sampling Locations	Geographic Coordinates	Annual Average Temperature (°C)	Annual Average Maximum Temperature (°C)	Annual Average Minimum Temperature (°C)	Annual Average Rainfall (mm)	Elevation (m)	Average Annual Sunshine Duration (h)
*P. perfoliatum*-1	Fujian Wuyishan	E 118°02′, N 27°39′	19	24	14	1926	350	1650.11
*P. perfoliatum*-2	Gangxi Hechi	E 107°43′, N 24°32′	20	25	17	1493	203	1372.76
*P. perfoliatum*-3	Anhui Bozhou	E 116°19′, N 33°31′	14.7	22	11	800	32	2141.76
*P. perfoliatum*-4	Zhejiang Taizhou	E 120°17′, N 28°14′	19.4	24	15	1632	141	1813.98
*P. perfoliatum*-5	Jiangxi Ganzhou	E 115°50′, N 26°05′	20.2	24	16	1447	107	1713.21
*P. perfoliatum*-6	Guizhou Guiyang	E 106°37′, N 26°21′	15.3	19	12	1130	1275	1173.17
*P. perfoliatum*-7	Guangdong Qingyuan	E 113°05′, N 24°26′	20.7	27	19	1900	105	1613.8
*P. perfoliatum*-8	Henan Luoyang	E 112°05′, N 34°10′	14.5	20	9	603	144	2151.33
*P. perfoliatum*-9	Guangxi Yizhou	E 24°09′, N 109°11′	20.4	26	17	1455.4	255	1696.9
*P. perfoliatum*-10	Guangdong Meizhou	E 115°58′, N 24°45′	21.2	28	18	1472.9	96	1847.24
*P. perfoliatum*-11	Fujian Fuzhou	E 118°46′, N 26°01′	19.7	24	16	1359	258	1677.31
*P. perfoliatum*-12	Yunnan Kunming	E 102°28, N 24°56′	15	23	11	924	1892	2173.98
*P. perfoliatum*-13	Hunan Changsha	E 114°10′, N 28°20′	17.2	21	14	1368	63	1577.08
*P. perfoliatum*-14	Hebei Baoding	E 114°55′, N 39°22′	13.4	18	8	498.9	338	2549.78
*P. perfoliatum*-15	Sichuan Chengdu	E 104°20, N 30°32′	16	20	13	904	500	1087.48

**Table 2:** Linear regression equations and *R*^2^ values of the standard curves for KP bioactive compounds.

Components	Linear Equation	Determination Coefficients (*R*^2^)
Gallic acid	Y = 53777X − 14524	*R*^2^ = 0.9992
Caffeic acid	Y = 36739X − 8649	*R*^2^ = 0.9991
Hyperoside	Y = 683275X − 438920	*R*^2^ = 0.9990
Quercetin	Y = 796966X − 634788	*R*^2^ = 0.9993
Isorhamnetin	Y = 550994X − 1.45741 × 10^6^	*R*^2^ = 0.9996

**Table 3:** Levels of KP bioactive compounds in *P. perfoliatum-x* (μg/g, *n* = 3).

The Source	Gallic Acid	Caffeic Acid	Hyperoside	Quercetin	Isorhamnetin
1	2.21 ± 0.10 ^a^	10.64 ± 0.31 ^f^	11.87 ± 0.34 ^d^	50.19 ± 1.04 ^b^	0.96 ± 0.02 ^e^
2	1.11 ± 0.03 ^e^	6.54 ± 0.26 ^g^	5.33 ± 0.16 ^f^	50.19 ± 1.09 ^b^	1.02 ± 0.01 ^d^
3	1.47 ± 0.05 ^c^	20.1 ± 0.65 ^d^	5.23 ± 0.12 ^f^	30.66 ± 0.63 ^d^	0.98 ± 0.02 ^d^
4	1.04 ± 0.04 ^e^	10.65 ± 0.33 ^f^	5.44 ± 0.14 ^f^	ND	1.01 ± 0.01 ^d^
5	1.24 ± 0.03 ^d^	23.3 ± 0.11 ^c^	3.22 ± 0.08 ^g^	23.82 ± 0.47 ^f^	1.43 ± 0.03 ^a^
6	ND	10.63 ± 032 ^f^	10.37 ± 0.30 ^e^	37.19 ± 0.76 ^c^	0.78 ± 0.02 ^f^
7	1.10 ± 0.04 ^e^	25.64 ± 0.99 ^b^	18.31 ± 0.54 ^a^	26.44 ± 0.44 ^e^	0.54 ± 0.01 ^h^
8	1.28 ± 0.05 ^d^	20.03 ± 0.81 ^d^	5.34 ± 0.11 ^f^	73.62 ± 1.48 ^a^	1.32 ± 0.02 ^b^
9	1.04 ± 0.03 ^e^	20.45 ± 0.83 ^c^	15.38 ± 0.41 ^b^	ND	0.65 ± 0.01 ^g^
10	0.62 ± 0.01 ^fg^	4.43 ± 0.11 ^h^	13.24 ± 0.35 ^c^	ND	ND
11	ND	10.37 ± 0.04 ^f^	17.43 ± 0.52 ^a^	24.09 ± 0.43 ^f^	0.41 ± 0.01 ^j^
12	0.54 ± 0.01 ^g^	28.35 ± 1.04 ^a^	ND	25.46 ± 0.51 ^ef^	0.48 ± 0.01 ^i^
13	0.74 ± 0.02 ^f^	20.36 ± 0.75 ^d^	15.89 ± 0.36 ^b^	15.99 ± 0.35 ^g^	0.66 ± 0.02 ^g^
14	0.49 ± 0.01 ^g^	15.43 ± 0.52 ^e^	12.34 ± 0.37 ^d^	15.67 ± 0.32 ^g^	0.53 ± 0.01 ^h^
15	1.60 ± 0.05 ^b^	18.64 ± 0.73 ^d^	9.85 ± 0.23 ^e^	30.66 ± 0.54 ^d^	1.24 ± 0.02 ^c^
CV (%)	43.2	43.7	50.0	50.0	38.2

Different lowercase letters indicate significance at the 0.05 level, $\bar{x}$ ± SD, μg/g; ND was excluded when performing the coefficient of variation (CV) calculations.

**Table 4:** Linear regression equations and *R*^2^ values of the standard curves for 13 inorganic elements.

Elements	Analytical Lines (nm)	Detection Limits (DL) µg/mL	Linear Equation	Determination Coefficients (*R*^2^)
Al	167.081	0.015	y = 95.422x + 33.836	*R*^2^ = 0.9998
As	228.812	0.002	y = 2127.9x + 514.61	*R*^2^ = 0.9999
Ba	230.424	0.002	y = 22133x + 1177	*R*^2^ = 0.9999
Ca	220.861	0.017	y = 19148x + 25226	*R*^2^ = 0.9993
Cr	267.716	0.0022	y = 15277x + 817.33	*R*^2^ = 1
Cu	324.754	0.0072	y = 26348x + 3810.9	*R*^2^ = 0.9999
Fe	238.204	0.0022	y = 19253x + 1375	*R*^2^ = 0.9999
Mg	285.213	0.004	y = 36173x + 2678	*R*^2^ = 0.9998
Mn	259.373	0.0071	y = 61869x + 1397.1	*R*^2^ = 1
Ni	221.647	0.0071	y = 10925x + 563.2	*R*^2^ = 1
Pb	220.353	0.0035	y = 1546.9x + 294.25	*R*^2^ = 1
Se	203.985	0.0028	y = 153.27x + 106.33	*R*^2^ = 1
Zn	213.856	0.0051	y = 8665x + 694.4	*R*^2^ = 0.9998

**Table 5:** Levels of 13 inorganic elements in *P. perfoliatum-x* (µg/g, *n* = 3).

Samples	Al	As	Ba	Ca	Cr	Cu	Fe	Mg	Mn	Ni	Pb	Se	Zn
1	34.8 ± 0.10 ^l^	6.5 ± 0.01 ^i^	9.2 ± 0.10 ^b^	631.0 ± 2.00 ^h^	4.9 ± 0.03 ^g^	0.5 ± 0.01 ^k^	18.0 ± 0.10 ^l^	113.0 ± 0.69 ^m^	37.8 ± 0.23 ^c^	0.6 ± 0.24 ^i^	1.2 ± 0.14	5.8 ± 0.43 ^l^	4.7 ± 0.42 ^h^
2	21.9 ± 0.10 ^n^	5.2 ± 0.01 ^j^	1.1 ± 0.05 ^m^	545.00 ± 3.00 ^j^	4.6 ± 0.02 ^h^	0.5 ± 0.02 ^l^	12.5 ± 0.30 ^m^	83.9 ± 1.31 ^n^	11.5 ± 0.13 ^g^	0.6 ± 0.13 ^i^	1.0 ± 0.17	4.9 ± 0.33 ^n^	2.8 ± 0.14 ^j^
3	54.1 ± 0.10 ^f^	9.3 ± 0.03 ^de^	6.4 ± 0.08 ^f^	765.0 ± 2.00 ^c^	4.8 ± 0.02 ^g^	3.0 ± 0.02 ^e^	39.9 ± 0.20 ^g^	172.0 ± 1.55 ^e^	0.0	0.9 ± 0.13 ^f^	1.7 ± 0.18	6.7 ± 0.23 ^g^	6.0 ± 0.48 ^d^
4	110.0 ± 0.2 ^b^	8.8 ± 0.03 ^e^	8.3 ± 0.08 ^c^	584.0 ± 4.00 ^i^	5.8 ± 0.01 ^e^	2.4 ± 0.003 ^h^	118.0 ± 0.32 ^c^	158.0 ± 1.50 ^i^	32.3 ± 0.10 ^d^	0.7 ± 0.10 ^h^	2.1 ± 0.10	6.7 ± 0.42 ^g^	5.4 ± 0.10 ^e^
5	49.7 ± 0.20 ^g^	8.5 ± 0.02 ^f^	6.6 ± 0.10 ^e^	643.0 ± 2.00 ^g^	5.0 ± 0.01 ^f^	2.2 ± 0.02 ^i^	37.0 ± 0.13 ^h^	153.0 ± 2.36 ^k^	51.3 ± 0.36 ^a^	0.8 ± 0.20 ^g^	1.7 ± 0.20	6.3 ± 0.10 ^j^	5.0 ± 0.45 ^f^
6	49.2 ± 0.10 ^h^	10.3 ± 0.03 ^c^	6.2 ± 0.10 ^g^	719.0 ± 1.00 ^e^	5.0 ± 0.01 ^f^	2.5 ± 0.02 ^g^	42.6 ± 0.33 ^f^	201.0 ± 1.75 ^c^	30.2 ± 0.21 ^e^	0.9 ± 0.32 ^f^	1.7 ± 0.11	6.4 ± 0.54 ^i^	4.3 ± 0.32 ^i^
7	49.0 ± 0.10 ^h^	8.9 ± 0.01 ^e^	6.3 ± 0.05 ^f^	722.0 ± 0.50 ^e^	4.9 ± 0.02 ^g^	2.7 ± 0.03 ^f^	36.2 ± 0.50 ^h^	156.0 ± 1.27 ^j^	50.7 ± 0.63 ^b^	0.9 ± 0.13 ^f^	1.7 ± 0.14	6.5 ± 0.63 ^h^	5.0 ± 0.40 ^f^
8	28.2 ± 0.30 ^m^	9.5 ± 0.07 ^d^	5.9 ± 0.03 ^h^	648.0 ± 2.00 ^g^	4.6 ± 0.02 ^h^	2.2 ± 0.02 ^i^	19.0 ± 0.25 ^k^	178.0 ± 2.23 ^d^	7.4 ± 0.11 ^h^	0.8 ± 0.10 ^g^	1.3 ± 0.17	5.6 ± 0.73 ^m^	2.1 ± 0.43 ^l^
9	47.2 ± 0.95 ^i^	7.2 ± 0.07 ^h^	3.1 ± 0.05 ^k^	780.0 ± 1.00 ^b^	5.9 ± 0.04 ^e^	4.7 ± 0.02 ^b^	17.9 ± 0.17 ^l^	170.0 ± 2.55 ^f^	12.7 ± 0.10 ^f^	4.9 ± 0.70 ^a^	2.7 ± 0.16	9.6 ± 0.41 ^a^	2.5 ± 0.34 ^k^
10	37.1 ± 0.63 ^k^	8.0 ± 0.10 ^g^	2.9 ± 0.06 ^l^	1200.0 ± 4.00 ^a^	6.0 ± 0.01 ^d^	2.0 ± 0.12 ^j^	23.3 ± 0.230 ^j^	166.0 ± 1.15 ^g^	3.7 ± 0.20 ^i^	1.2 ± 0.23 ^b^	1.8 ± 0.43	6.1 ± 0.56 ^k^	2.5 ± 0.24 ^k^
11	72.6 ± 0.73 ^d^	7.9 ± 0.10 ^g^	4.1 ± 0.04 ^j^	741.0 ± 1.00 ^d^	6.0 ± 0.02 ^d^	4.9 ± 0.03 ^a^	61.4 ± 0.21 ^d^	134.0 ± 1.52 ^l^	0.0	1.1 ± 0.13 ^c^	2.4 ± 0.78	9.5 ± 0.68 ^b^	9.2 ± 0.65 ^b^
12	109.0 ± 0.70 ^c^	9.5 ± 0.05 ^d^	6.5 ± 0.05 ^e^	734.0 ± 3.00 ^d^	6.4 ± 0.03 ^b^	3.6 ± 0.02 ^d^	190.0 ± 0.31 ^a^	163.0 ± 1.98 ^h^	0.0	1.0 ± 0.21 ^de^	2.8 ± 0.45	9.4 ± 0.44 ^c^	6.7 ± 0.41 ^c^
13	65.4 ± 0.10 ^e^	11.1 ± 0.10 ^b^	10.7 ± 0.05 ^a^	696.0 ± 3.00	6.0 ± 0.002 ^d^	3.8 ± 0.01 ^c^	50.0 ± 0.90 ^e^	230.0 ± 1.63 ^b^	0.0	1.0 ± 0.10 ^d^	3.6 ± 0.61	7.5 ± 0.31 ^f^	4.8 ± 0.61 ^g^
14	115.0 ± 0.20 ^a^	9.0 ± 0.10 ^e^	5.4 ± 0.02 ^i^	504.0 ± 2.00 ^k^	8.7 ± 0.03 ^a^	2.0 ± 0.02 ^j^	128.0 ± 0.63 ^b^	166.0 ± 2.01 ^g^	0.0	1.0 ± 0.14 ^e^	2.4 ± 0.32	8.2 ± 0.49 ^d^	5.5 ± 0.47 ^e^
15	41.1 ± 0.10 ^j^	11.8 ± 0.10 ^a^	7.2 ± 0.02 ^d^	775.0 ± 1.00 ^b^	6.2 ± 0.01 ^c^	2.3 ± 0.03 ^i^	27.6 ± 0.31 ^i^	257.0 ± 1.10 ^a^	0.0	1.1 ± 0.15 ^c^	2.1 ± 0.10	7.7 ± 0.89 ^e^	10.8 ± 0.72 ^a^
average	58.95	8.77	5.98	712.47	5.66	2.61	54.76	166.73	15.84	1.16	2.02	7.13	5.14
CV (%)	50.92	26.4	42.1	28.7	28.9	48.2	75.5	29.4	130	90.5	34.0	20.7	51.1

Different lowercase letters indicate significance at the 0.05 level, $\bar{x}$ ± SD, μg/g.

**Table 6:** Pearson’s correlation coefficients between KP bioactive compounds and inorganic elements.

	Gallic Acid	Caffeic Acid	Hyperoside	Quercetin	Isorhamnetin
Al	−0.425	0.214	−0.137	−0.590 *	−0.291
As	−0.168	0.445	−0.005	−0.215	0.112
Ba	0.3	0.356	−0.017	−0.146	0.281
Ca	−0.16	−0.176	0.253	−0.335	−0.557 *
Cr	−0.393	0.025	0.184	−0.522 *	−0.441
Cu	−0.508	0.404	0.323	−0.229	−0.358
Fe	−0.401	0.296	−0.354	−0.444	−0.274
Mg	−0.1	0.35	0.131	−0.192	0.074
Mn	0.282	0.089	0.008	0.034	0.314
Ni	−0.054	0.174	0.32	0.074	−0.202
Pb	−0.434	0.372	0.275	−0.453	−0.396
Se	−0.433	0.376	0.24	−0.28	−0.41
Zn	−0.053	0.181	0.047	−0.277	0.062

* *p* < 0.05.

**Table 7:** Pearson’s correlation coefficients between KP bioactive compounds and climatic parameters in *P. perfoliatum-x*.

	Annual Average Temperature	Annual Average Maximum Temperature	Annual Average Minimum Temperature	Annual Average Rainfall	Elevation	Average Annual Sunshine Duration
Gallic acid	0.101	0.135	0.036	0.226	−0.37	−0.072
Caffeic acid	−0.298	−0.143	−0.221	−0.238	0.238	0.252
Hyperoside	0.354	0.209	0.411	0.364	−0.372	−0.24
Quercetin	−0.359	−0.354	−0.379	−0.228	0.088	−0.086
Isorhamnetin	−0.2	−0.363	−0.256	−0.14	−0.172	−0.21

**Table 8:** Pearson’s correlation coefficients between inorganic elements and climatic elements in *P. perfoliatum-x*.

	Annual Average Temperature	Annual Average Maximum Temperature	Annual Average Minimum Temperature	Annual Average Rainfall	Elevation	Average Annual Sunshine Duration
Al	−0.331	−0.266	−0.364	−0.263	0.349	0.531 *
As	−0.578 *	−0.607 *	−0.45	−0.488	0.24	−0.073
Ba	−0.294	−0.383	−0.302	0.064	0.053	−0.022
Ca	0.309	0.471	0.377	0.12	−0.013	−0.114
Cr	−0.337	−0.337	−0.358	−0.426	0.176	0.445
Cu	−0.009	0.035	0.056	−0.135	0.131	0.105
Fe	−0.403	−0.245	−0.42	−0.32	0.594 *	0.528 *
Mg	−0.444	−0.508 *	−0.316	−0.422	0.132	−0.2
Mn	0.47	0.328	0.426	0.648 **	−0.123	−0.28
Ni	0.242	0.267	0.244	0.062	−0.048	−0.024
Pb	−0.164	−0.175	−0.104	−0.164	0.208	0.136
Se	−0.145	−0.086	−0.113	−0.23	0.367	0.211
Zn	−0.22	−0.286	−0.141	−0.177	0.216	−0.197

* The correlation is significant at the 0.05 level (two-tailed); ** The correlation is significant at the 0.01 level (two-tailed).

## Data Availability

Data is contained within the article and Supplementary Materials.

## Supplementary Materials

The following supporting information can be downloaded at: https://www.mdpi.com/article/10.3390/molecules30102231/s1, Table S1: Eigenvalues, variance contribution rates, and the cumulative variance contribution rate of PC1 and PC2; Table S2: A component score coefficient matrix showing the correlations of PC1 and PC2 with the levels of KP bioactive compounds in *P. perfoliatum-x*; Table S3: Eigenvalues, variance contribution rates, and the cumulative variance contribution rate of PC1, PC2, PC3, and PC4; Table S4: A component score coefficient matrix showing the correlations of PC1, PC2, PC3, and PC4 with the levels of inorganic elements in *P. perfoliatum-x*; Table S5: Recovery by sample spiking content Comparison; Table S6: Precision content Comparison; Table S7: Repeatability content Comparison; Table S8: Stability content Comparison.

## References

[1] Committee N.P. (2020). Pharmacopoeia of the People’s Republic of China.

[2] Liu J., Zeng Y., Sun G., Yu S., Xu Y., He C., Li Z., Jin S., Qin X. (2020). *Polygonum perfoliatum* L., an Excellent Herbal Medicine Widely Used in China: A Review. Front. Pharmacol..

[3] Lei J., Yao N., Wang K.-W. (2013). Phytochemical and chemotaxomic study on *Polygonum perfoliatum* L.. Biochem. Syst. Ecol..

[4] Wang K.W., Zhu J.R., Shen L.Q. (2013). A new lignan with anti-tumour activity from *Polygonum perfoliatum* L.. Nat. Prod. Res..

[5] Cheng H.B., Liu X.Q., Chen K.L. (2012). Chemical constituents of ethyl acetate extract from *Polygonum perfoliatum*. Zhong Yao Cai.

[6] Fan D., Zhao Y., Zhou X., Gong X., Zhao C. (2014). Simultaneous determination of esculetin, quercetin-3-O-β-D-glucuronide, quercetin-3-O-β -D-glucuronopyranside methyl ester and quercetin in effective part of *Polygonum perfoliatum* L. using high performace liquid chromatography. Pharmacogn. Mag..

[7] Liu X.-X. (2008). Study on Effective Composition Analysis and Antibacterial Effects of Herb *Polygonum perfoliatum*. Prog. Vet. Med..

[8] Liu P., Tang X., Gong C., Xu G. (2010). Manganese tolerance and accumulation in six Mn hyperaccumulators or accumulators. Plant Soil.

[9] Xue S., Wang J., Wu C., Li S., Hartley W., Wu H., Zhu F., Cui M. (2018). Physiological response of *Polygonum perfoliatum* L. following exposure to elevated manganese concentrations. Environ. Sci. Pollut. Res..

[10] Shen L., Pang S., Zhong M., Sun Y., Qayum A., Liu Y., Rashid A., Xu B., Liang Q., Ma H. (2023). A comprehensive review of ultrasonic assisted extraction (UAE) for bioactive components: Principles, advantages, equipment, and combined technologies. Ultrason. Sonochem..

[11] Zhang J., Yu X., Yang R., Zheng B.Q., Zhang Y.Q., Zhang F. (2024). Quality evaluation of Lonicerae Japonicae Flos from different origins based on high-performance liquid chromatography (HPLC) fingerprinting and multicomponent quantitative analysis combined with chemical pattern recognition. Phytochem. Anal..

[12] Mrmošanin J., Pavlović A., Rašić Mišić I., Tošić S., Petrović S., Mitić Z., Pecev-Marinković E., Arsić B. (2024). Evaluation of an Inductively Coupled Plasma–Atomic Emission Spectrometry (ICP-AES) Method for the Determination of Macro and Microelements in *Trifolium* L. Species. Anal. Lett..

[13] Xu D., Huang L., Chen Y., Ye S., Tang Y. (2021). Research Progress on Chemical Constituents, Pharmacological Action and Quality Standard of *Polygonum perfoliatum* L.. Chin. Wild Plant Resour..

[14] Long S., Zao Y., Zhou X., Gong X., Chen H., Zhao C. (2011). Determination of Heavy Metal in *Polygonum perfoliatum* and Evaluation on its Quality. Chin. J. Exp. Tradit. Med. Formulae.

[15] Huang G., Zhao W., Liao Z., Wang J., Xu L. (2022). Determination of Content of Total Flavonoids in Polygonum perfoliatum from Different Areas. Guangzhou Chem. Ind..

[16] Zhang D., Zhang X., Li Y., Huang H., Lin S., Liu H. (2018). Determination and Comparison of Content of Trace Elements in *Polygonum perfoliatum* L. from Different Origins. J. Anhui Agric. Sci..

[17] Pan Y., Zhang H., Zhang P., Pei S., Fan J., Liu S. (2021). Prediction of Suitable Distribution Area of Polygonum perfoliatum L. Based on MaxEnt Model and ArcGIS. Chin. J. Inf. Tradit. Chin. Med..

[18] Sun X., Chen H., Zhou X. (2017). Research progress on chemical composition, quality control and pharmacological action of *Polygonum perfoliatum* L.. Shandong Med. J..

[19] Huang Y., Shi T., Luo X., Xiong H., Min F., Chen Y., Nie S., Xie M. (2019). Determination of multi-pesticide residues in green tea with a modified QuEChERS protocol coupled to HPLC-MS/MS. Food Chem..

[20] Chiang M.C., Tsai T.Y., Wang C.J. (2023). The Potential Benefits of Quercetin for Brain Health: A Review of Anti-Inflammatory and Neuroprotective Mechanisms. Int. J. Mol. Sci..

[21] Gao J., Hu J., Hu D., Yang X. (2019). A Role of Gallic Acid in Oxidative Damage Diseases: A Comprehensive Review. Nat. Prod. Commun..

[22] Xie Y., Huang B., Yu K., Shi F., Liu T., Xu W. (2013). Caffeic acid derivatives: A new type of influenza neuraminidase inhibitors. Bioorganic Med. Chem. Lett..

[23] Plaper A., Golob M., Hafner I., Oblak M., Solmajer T., Jerala R. (2003). Characterization of quercetin binding site on DNA gyrase. Biochem. Biophys. Res. Commun..

[24] Samoilova Z., Tyulenev A., Muzyka N., Smirnova G., Oktyabrsky O. (2019). Tannic and gallic acids alter redox-parameters of the medium and modulate biofilm formation. AIMS Microbiol..

[25] Zhang J., Fu H., Xu Y., Niu Y., An X. (2016). Hyperoside reduces albuminuria in diabetic nephropathy at the early stage through ameliorating renal damage and podocyte injury. J. Nat. Med..

[26] Wang Y., Wang X., Chen X., Xu W., Wu Y. (2018). Fingerprints Study on the Epimedium acuminaum Franch.in Different Areas of Guizhou Province Based on Principal. Seed.

[27] Li M., Zhang Y., Xi H., Fu Y., Wang H., Zhang Y., Sun S. (2022). Characterization of Rose Essential Oils by Double-Region Atmospheric Pressure Chemical Ionization Mass Spectrometry (DRAPCI-MS) with Principal Component Analysis (PCA), Hierarchical Cluster Analysis (HCA), and Heatmap Analysis. Anal. Lett..

[28] (2014). Proceedings of the 11th International Conference on Innovation and Management.

[29] Zhou H., Liu F., Zhang X. (2022). Study on climate regionalization of Longtan pearl plum planting in Hechi City based on GIS. Hubei Agric. Sci..

[30] Li Y., Lu X. (2024). Research on the Issues and Countermeasures of Mulberry Silkworm Industry in Yizhou District, Hechi City. Guangdong Seric..

[31] Kalogiouri N.P., Manousi N., Zachariadis G.A. (2021). Determination of the Toxic and Nutrient Element Content of Almonds, Walnuts, Hazelnuts and Pistachios by ICP-AES. Separations.

[32] Wu H., Chang X., Sang X., Qu B., Cui H., Peng X. (2018). Comprehensive evaluation of twenty-seven varieties of mineral elements in Tetrastigma hemsleyanum from different growing areas. Chin. Tradit. Pat. Med..

[33] Feng W., Kita D., Peaucelle A., Cartwright H.N., Doan V., Duan Q., Liu M.C., Maman J., Steinhorst L., Schmitz-Thom I. (2018). The FERONIA Receptor Kinase Maintains Cell-Wall Integrity during Salt Stress through Ca(2+) Signaling. Curr. Biol..

[34] Dandan M. (2011). Functional Analyses of AtMGT6 in Mg-Transport in *Arabidopsis*. Ph.D. Thesis.

[35] Koletzko B., Goulet O., Hunt J., Krohn K., Shamir R. (2005). 1. Guidelines on Paediatric Parenteral Nutrition of the European Society of Paediatric Gastroenterology, Hepatology and Nutrition (ESPGHAN) and the European Society for Clinical Nutrition and Metabolism (ESPEN), Supported by the European Society of Paediatric Research (ESPR). J. Pediatr. Gastroenterol. Nutr..

[36] Lötscher J., Martí I.L.A.A., Kirchhammer N., Cribioli E., Giordano Attianese G.M.P., Trefny M.P., Lenz M., Rothschild S.I., Strati P., Künzli M. (2022). Magnesium sensing via LFA-1 regulates CD8(+) T cell effector function. Cell.

[37] Li C., Lei X., Che S. (2012). Coordination bonding based pH-responsive albumin nanoparticles for anticancer drug delivery. Dalton Trans..

[38] Ahamed M., Khan M.A.M. (2023). Enhanced Photocatalytic and Anticancer Activity of Zn-Doped BaTiO_3_ Nanoparticles Prepared Through a Green Approach Using Banana Peel Extract. Catalysts.

[39] Schümann K., Ettle T., Szegner B., Elsenhans B., Solomons N.W. (2007). On risks and benefits of iron supplementation recommendations for iron intake revisited. J. Trace Elem Med. Biol..

[40] Dambiec M., Polechońska L., Klink A. (2013). Levels of essential and non-essential elements in black teas commercialized in Poland and their transfer to tea infusion. J. Food Compos. Anal..

[41] Zuo T., Shen M., Zhang L., Jin H., Ma S. (2023). Formulation of limit standards for heavy metals and harmful elements in TCMs and related reflections. Chin. J. Pharm. Anal..

[42] Vitali D., Vedrina Dragojević I., Šebečić B. (2008). Bioaccessibility of Ca, Mg, Mn and Cu from whole grain tea-biscuits: Impact of proteins, phytic acid and polyphenols. Food Chem..

[43] World Health Organization (2007). WHO Guidelines for Assessing Quality of Herbal Medicines with Reference to Contaminants and Residues.

[44] Liu P., Wang C.N., Song X., Wu Y.N. (2010). Dietary intake of lead and cadmium by children and adults—Result calculated from dietary recall and available lead/cadmium level in food in comparison to result from food duplicate diet method. Int. J. Hyg. Environ. Health.

[45] Li M., Liu Y., Zhou R., Lin Q., Wu B. (2007). Analysis on Limit Standards for Heavy Metals and Arsenic Salts in Traditional Chinese Medicine Both at Home and Abroad. Lishizhen Med. Mater. Medica Res..

[46] Zoroddu M.A., Aaseth J., Crisponi G., Medici S., Peana M., Nurchi V.M. (2019). The essential metals for humans: A brief overview. J. Inorg. Biochem..

[47] Prihantono, Irfandi R., Raya I. (2020). Warsinggih, Potential anticancer activity of Mn (II) complexes containing arginine dithiocarbamate ligand on MCF-7 breast cancer cell lines. Ann. Med. Surg..

[48] Wang M., Li M., Wang Y., Shao Y., Zhu Y. (2021). Efficient Antibacterial Activity of Hydroxyapatite through ROS Generation Motivated by Trace Mn(III) Coupled H Vacancy. J. Mater. Chem. B.

[49] Stanojković-Sebić A., Maksimović J., Dinić Z., Poštić D., Ilićić R., Stanojković A. (2017). Microelements and Heavy Metals Content in Frequently Utilized Medicinal Plants Collected from the Power Plant Area. Nat. Prod. Commun..

[50] Köhrle J. (2021). Selenium in Endocrinology-Selenoprotein-Related Diseases, Population Studies, and Epidemiological Evidence. Endocrinology.

[51] Wang S., Kang X., Dai J., Dai W., Zhang J., Ji J. (2023). Evaluation of Areca Quality Based on Principal Component and Hierarchical Cluster Analyses in Hainan, China. HortScience.

[52] Singh D. (2019). Principal Component Analysis in Bitter Gourd (*Momordica charantia* L.). Bangladesh J. Bot..

[53] Zhang Q., Ma X., Wei W. (2017). Evaluation of climate comfort of human settlements in Meizhou city. Guangdong Meteorol..

[54] Zhao Q., Zhao X., Huang P., Pu J., Zhou S., Feng Y., Gu Z., Shi X., Chu B. (2024). Soil Carbon Changes and Its Influencing Factors in Major Forest Types in the Subtropical Area of Yunnan Province. For. Res..

[55] Yang Q., You X., Zhang H., Mwenda K., Wang Y., Huang Y. (2020). A New Method to Predict Erythrocyte Sedimentation Rate with Natural Geographical Factors and Location by Case-based Reasoning: A Case Study of China. Chin. Geogr. Sci..

[56] Lu Y., Xin Y., Li J., Wang J. (2024). Geochronological and Geochemical Constraints of Leucogranites inJianning Area, Central Wuyishan, and Their Geological Implications. Acta Geosci. Sin..

[57] Li J., Zhang M., Wang J., Fu Z. (1999). Effects of Isorhamnetin on intracelluar free calcium concentration in cultured rabbit aortic smooth muscle cells. J. Southwest Med. Univ..

[58] Liu G., Li D., Mai H., Lin X., Lu X., Chen K., Wang R., Riaz M., Tian J., Liang C. (2024). GmSTOP1-3 regulates flavonoid synthesis to reduce ROS accumulation and enhance aluminum tolerance in soybean. J. Hazard. Mater..

[59] Pandey N., Hasanuzzaman M., Nahar K., Fujita M. (2018). Antioxidant Defense System in Plants Exposed to Metal Toxicity. Plants Under Metal and Metalloid Stress: Responses, Tolerance and Remediation.

[60] Lv M., Chen M., Zhang R., Zhang W., Wang C., Zhang Y., Wei X., Guan Y., Liu J., Feng K. (2020). Manganese is critical for antitumor immune responses via cGAS-STING and improves the efficacy of clinical immunotherapy. Cell Res..

[61] Al Zahrani N.A., El-Shishtawy R.M., Asiri A.M. (2020). Recent developments of gallic acid derivatives and their hybrids in medicinal chemistry: A review. Eur. J. Med. Chem..

[62] Prasad N.R., Karthikeyan A., Karthikeyan S., Reddy B.V. (2011). Inhibitory effect of caffeic acid on cancer cell proliferation by oxidative mechanism in human HT-1080 fibrosarcoma cell line. Mol. Cell. Biochem..

[63] Choudhary N., Collignon T.E., Tewari D., Bishayee A. (2022). Hypericin and its anticancer effects: From mechanism of action to potential therapeutic application. Phytomedicine.

[64] Bhosale P.B., Ha S.E., Vetrivel P., Kim H.H., Kim S.M., Kim G.S. (2020). Functions of polyphenols and its anticancer properties in biomedical research: A narrative review. Transl. Cancer Res..

[65] Biswas P., Abu Kaium M., Tareq M.M.I., Tauhida S.J., Hossain M.R., Siam L.S., Parvez A., Bibi S., Hasan M.H., Rahman M.M. (2024). The experimental significance of isorhamnetin as an effective therapeutic option for cancer: A comprehensive analysis. Biomed. Pharmacother..

[66] Km C. (2002). Dietary Flavonoid Quercetin in Relation to Cataract. Ph.D. Thesis.

[67] Milner M.J., Seamon J., Craft E., Kochian L.V. (2013). Transport properties of members of the ZIP family in plants and their role in Zn and Mn homeostasis. J. Exp. Bot..

[68] Lee M.Y., Ojeda-Britez S., Ehrbar D., Samwer A., Begley T.J., Melendez J.A. (2022). Selenoproteins and the senescence-associated epitranscriptome. Exp. Biol. Med..

[69] Dong J.Z., Ding J., Yu P.Z., Lei C., Zheng X.J., Wang Y. (2013). Composition and distribution of the main active components in selenium-enriched fruit bodies of Cordyceps militaris link. Food Chem..

[70] Kostic D., Velickovic J., Mitic S., Mitić M., Randjelovic S., Arsic B., Pavlović A. (2012). Correlation among phenolic, toxic metals and antioxidant activity of the extracts of plant species from Southeast Serbia. Bull. Chem. Soc. Ethiop..

[71] Tokalıoğlu Ş. (2012). Determination of trace elements in commonly consumed medicinal herbs by ICP-MS and multivariate analysis. Food Chem..

[72] Chen L., Chang L., Cao F., Wang G., Dong X. (2013). Effects of Temperature and Soil Water Deficit on the Flavonoid Content and Activities of Enzymes Involved in Ginkgo Leaves. Acta Bot. Boreali-Occident. Sin..

[73] Wang G., Cao F., Chang L., Guo X., Wang J. (2014). Temperature has more effects than soil moisture on biosynthesis of flavonoids in Ginkgo (*Ginkgo biloba* L.) leaves. New For..

[74] Meng J.-F., Ning P.-F., Xu T.-F., Zhang Z.-W. (2013). Effect of Rain-Shelter Cultivation of Vitis vinifera cv. Cabernet Gernischet on the Phenolic Profile of Berry Skins and the Incidence of Grape Diseases. Molecules.

[75] Zhang X., Zhou T., Guo L., Zhu S., Huang L. (2011). Ecology suitability of Polygonum capitatum in Guizhou province based on topographical conditions. Zhongguo Zhong Yao Za Zhi.

[76] Min R. (2018). Effects of Water Stress on Tannin Content and Flavonoid Content of *Lespedeza davurica*. Master’s Thesis.

[77] Salami M., Heidari B., Batley J., Wang J., Tan X.-L., Richards C., Tan H. (2024). Integration of genome-wide association studies, metabolomics, and transcriptomics reveals phenolic acid-and flavonoid-associated genes and their regulatory elements under drought stress in rapeseed flowers. Front. Plant Sci..

[78] Oruc H., Sorucu A., Ünal H., Aydin L. (2017). Effects of season and altitude on biological active certain phenolic compounds levels and partial standardization of propolis. Vet. J. Ank. Univ..

[79] Sardans J., Peñuelas J., Prieto P., Estiarte M. (2008). Changes in Ca, Fe, Mg, Mo, Na, and S content in a Mediterranean shrubland under warming and drought. J. Geophys. Res. Biogeosci..

[80] Feng J., Zhou Y., Bai Y., Fan M., Wang Y., Tang F., Feng J. (2025). Changes in rainfall impact the release of metal elements in the litter of a subtropical mixed forest. Environ. Res..

[81] Zhai Y., Li R., Zhang Q., Qi M., Lu B., Huang L., Xu X. (2024). Effects of Temperature and Arsenic on Growth and Arsenic Uptake of Different Rice Varieties during Seedlings Stage. Acta Pedol. Sin..

[82] Kabata-Pendias A. (2010). Trace Elements in Soils and Plants.

[83] Xu L., Lu A., Wang J. (2016). Research Progress on the Effects of Temperature Changes on the Availability of Heavy Metals in Plants. Jiangsu Agric. Sci..

[84] Ma J. (2004). Laws of Soil Vertical Variations on Southern Slope of Funiu Mt.: Simultaneous Study on Subtropical Zone. Acta Geogr. Sin..

[85] Montgomery B.L., Oh S., Karakkat B. (2015). Molecular basis and fitness implications of the interplay between light and the regulation of iron homeostasis in photosynthetic organisms. Environ. Exp. Bot..

[86] Lingyan Z., Yubin Z., Zonggeng L., Wenke L. (2019). Effect of Continuous Red/Blue LED Light and Its Light Intensity on Growth and Mineral Elements Absorption of Lettuce. Spectrosc. Spectr. Anal..

[87] Sharma M., Kothari C., Sherikar O., Mehta P. (2014). Concurrent estimation of amlodipine besylate, hydrochlorothiazide and valsartan by RP-HPLC, HPTLC and UV-spectrophotometry. J. Chromatogr. Sci..

[88] Harshit D., Charmy K., Nrupesh P. (2017). Organophosphorus pesticides determination by novel HPLC and spectrophotometric method. Food Chem..

[89] Kowalska M., Woźniak M., Kijek M., Mitrosz P., Szakiel J., Turek P. (2022). Management of validation of HPLC method for determination of acetylsalicylic acid impurities in a new pharmaceutical product. Sci. Rep..

[90] Pereira J.B., Dantas K.G.F. (2016). Evaluation of inorganic elements in cat’s claw teas using ICP OES and GF AAS. Food Chem..

[91] Du Q., Cai Y., Chen Z., Wei D., Cao Y., Chen Y., Yu S., Zhao Q., Wu J., Liu M. (2020). Determination of Trace Elements in Corydalis conspersa and Corydalis linarioides by ICP-AES. J. Chem..

