# Supplementary material for: Impact of Geographical Origin on the Contents of Inorganic Elements and Bioactive Compounds in Polygonum perfoliatum L
Source: Molecules. 2025 May 21;30(10):2231. doi: 10.3390/molecules30102231 (PMC12113674; doi:10.3390/molecules30102231)
Supplement: Supplementary file 1 [file molecules-30-02231-s001.zip › molecules-3614253-supplementary.pdf]

Table S1. Eigenvalues, variance contribution rates, and the cumulative variance contribution rate of PC1 and PC2.

PC	Eigenvalue	Variance contribution rate (%)	Cumulative variance contribution (%)
1	2.578	51.557	51.557
2	1.055	21.106	72.664

Table S2 A component score coefficient matrix showing the correlations of PC1 and PC2 with the levels of KP bioactive compounds in *P.perfoliatum*-x.

Components	PC1	PC2
Isorhamnetin	0.867	0.449
Hyperoside	-0.809	-0.350
Quercetin	0.730	-0.317
Gallic acid	0.683	-0.178
Caffeic acid	-0.413	0.774

Table S3. Eigenvalues, variance contribution rates, and the cumulative variance contribution rate of PC1, PC2, PC3, and PC4.

PC	Eigenvalue	Variance contribution rate (%)	Accumulative variance contribution (%)
1	4.960	38.151	38.151
2	2.342	18.013	56.164
3	2.070	15.922	72.086
4	1.121	8.625	80.711

Table S4. A component score coefficient matrix showing the correlations of PC1, PC2, PC3, and PC4 with the levels of inorganic elements in *P.perfoliatum*-x.

Elements	PC1	PC2	PC3	PC4
Pb	0.883	-0.166	0.061	0.202
Se	0.857	-0.353	-0.199	0.145
Al	0.740	0.319	-0.519	0.054
Cr	0.728	0.023	-0.359	-0.303
Cu	0.713	-0.469	0.106	0.303
Fe	0.663	0.343	-0.536	-0.056
Zn	0.550	0.327	0.119	-0.210
Ni	0.240	-0.812	0.013	0.385
Ba	0.314	0.681	0.370	0.382
Ca	0.037	-0.527	0.449	-0.348
Mg	0.580	0.104	0.748	-0.047
As	0.608	0.364	0.646	-0.059
Mn	-0.505	0.314	-0.028	0.636

Table S5. Recovery by sample spiking content Comparison

serial number	Gallic acid	Caffeic acid	Hypericin	Quercetin	Isorhamnetin
1	2.48	12.08	12.53	100.57	2.15
2	2.37	12.13	12.55	100.56	2.15
3	2.29	12.02	12.67	100.67	2.16
Average Recovery Rate	94.90%	87.41%	104.18%	91.64%	95.03%

Table S6. Precision content Comparison

serial number	Gallic acid	Caffeic acid	Hypericin	Quercetin	Isorhamnetin
1	2.23	10.53	12.47	50.42	1.07
2	2.21	10.48	12.07	49.98	1.06
3	2.20	10.40	11.99	50.52	1.07
4	2.32	10.48	11.91	51.00	1.06
5	2.20	11.15	11.92	50.73	1.06
6	2.27	10.76	11.75	49.83	1.07
RSD	2.24%	2.26%	2.21 %	0.88%	0.51%

Table S7. Repeatability content Comparison

serial number	Gallic acid	Caffeic acid	Hypericin	Quercetin	Isorhamnetin
1	2.28	10.53	11.70	48.92	1.06
2	2.31	10.10	12.10	49.98	1.07
3	2.20	10.44	12.30	50.52	1.06
4	2.29	10.48	11.76	50.99	1.07
5	2.20	10.77	11.95	49.73	1.07
6	2.27	10.76	12.33	49.83	1.07
RSD	2.20%	2.63%	2.20 %	0.87%	0.48%

Table S8. Stability content Comparison

times	Gallic acid	Caffeic acid	Hypericin	Quercetin	Isorhamnetin
0h	2.48	12.08	12.53	100.57	2.15
2h	2.48	12.13	12.55	100.56	2.15
4h	2.49	12.02	12.67	100.67	2.16
8h	2.47	12.29	12.52	100.55	2.15
10h	2.56	12.36	12.56	100.77	2.15
12h	2.47	12.34	12.57	101.08	2.14
24h	2.54	12.29	12.65	100.70	2.15
48h	2.51	12.19	12.69	101.78	2.15
RSD	1.40%	1.13%	0.54%	0.42%	0.25%
